# Supplementary material for: Photoluminescence upconversion in monolayer WSe2 activated by plasmonic cavities through resonant excitation of dark excitons
Source: Nat Commun. 2023 Sep 15;14:5726. doi: 10.1038/s41467-023-41401-8 (PMC10504321; doi:10.1038/s41467-023-41401-8)
Supplement: Supplementary file 1 — Supplementary Information [file 41467_2023_41401_MOESM1_ESM.pdf]

Supplementary Information

Photoluminescence Upconversion in Monolayer WSe_2 Activated by Plasmonic Cavities through Resonant Excitation of Dark Excitons

Niclas S. Mueller, Rakesh Arul, Gyeongwon Kang, Ashley P. Saunders, Amalya C. Johnson, Ana Sánchez-Iglesias, Shu Hu, Lukas A. Jakob, Jonathan Bar-David, Bart de Nijs, Luis M. Liz-Marzán, Fang Liu, Jeremy J. Baumberg

Index to contents of Supplementary Information:

Section S1: PL spectra of WSe_2 on Au vs glass substrate
Section S2: Spatial localization of PL enhancement
Section S3: Excitation power dependence of anti-Stokes PL
Section S4: Estimate of local experimental enhancement factors by NPoM cavities
Section S5: Estimate of PL efficiency and quantum yield
Section S6: Statistics of dark exciton activation by plasmonic cavities and peak fits
Section S7: FDTD simulation of PL enhancement
Section S8: Electrochemical gating of PL emission

Section S1: PL spectra of WSe_2 on Au vs glass substrate

To prevent quenching of the PL emission by the gold substrates we used a special approach of 1. exfoliating WSe_2 monolayers with the gold tape method, 2. transferring the monolayers to an insulating substrate, and 3. transferring the monolayers back to a template-stripped Au substrate (see Methods). The PL intensities of WSe_2 are compared in Figure S1. The PL intensity of the back-transferred WSe_2 is only decreased by a factor x2 (peak area) compared to WSe_2 on a CaF_2 substrate indicating minimum quenching by the gold surface (Fig. S1, compare blue and orange). The PL emission of WSe_2 in direct contact with Au after exfoliation is on the other hand completely quenched (Fig. S1, black). We attribute the much larger PL after the back-transfer to polymer residues from the transfer process that decouple the WSe_2 monolayer from the Au substrate. This works reproducibly over large areas, as can be seen from the intensity of reference spectra recorded at 250 random positions on the substrate (Fig. S5b).

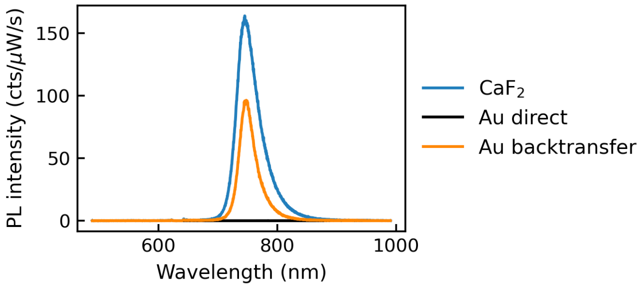

Figure S1: PL spectrum of WSe_2 on Au vs glass. Stokes PL spectra recorded with λ_l = 633 nm of monolayer WSe_2 on CaF_2 (blue), in direct contact with Au after exfoliation (black), and after subsequent transfer to an insulating substrate and back onto an Au surface (orange).

Section S2: Spatial localization of PL enhancement

To determine the spatial localization of the PL enhancement, we focus a λ_l = 785 nm CW excitation laser to a diffraction-limited spot with a 100x 0.9NA objective. The spectrometer slit width is set to 200 µm and readout lines on the CCD are limited for confocal detection. We scan the laser excitation spot across 4 NPoM cavities with a step size of 50 nm. Similar to NDoMs, Figs. 1e, the enhancement is tightly localized to the NPoM cavities (Fig. S2a, b). From fits with Gaussians we extract a FWHM of the enhanced PL signal of 460 ± 30 nm on average (Fig. S2a, c). This is close to the diffraction limited spot size $\lambda_l/2\mathrm{NA}$ = 440 nm of the excitation laser.

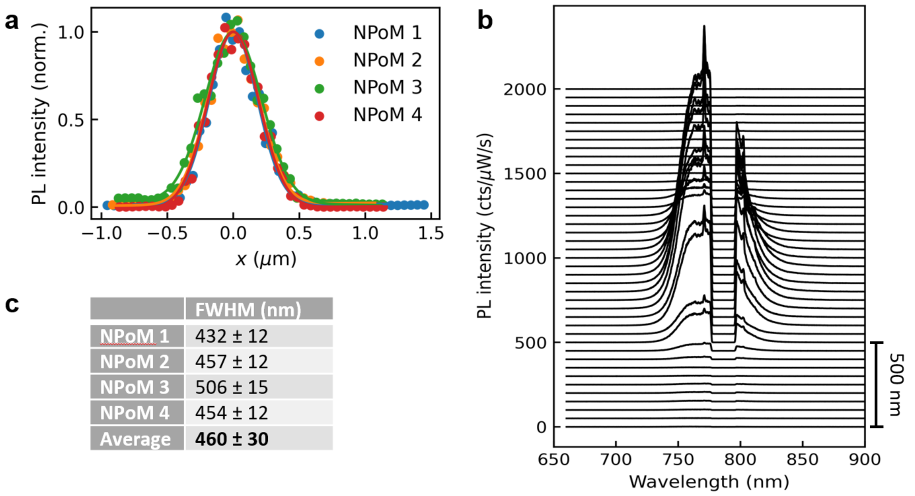

	FWHM (nm)
NPoM 1	432 ± 12
NPoM 2	457 ± 12
NPoM 3	506 ± 15
NPoM 4	454 ± 12
Average	**460 ± 30**

Figure S2: Spatial localization of PL enhancement by plasmonic NPoM cavities. (a) Normalized anti-Stokes PL intensity of WSe_2 when scanning λ_l = 785 nm excitation laser spot across four NPoM cavities. Data are fit with Gaussian peaks and centred on *x* axis. (b) PL spectra enhanced by NPoM cavity when scanning excitation laser across NPoM cavity (bottom to top). Narrow peaks on top of the PL spectra are resonant surface-enhanced Raman scattering from WSe_2. (c) Full-width at half-maxima (FWHM) of the Gaussian peaks fitted to the line scans in (a).

Section S3: Excitation power dependence of anti-Stokes PL

To better understand the mechanism of anti-Stokes PL, we measure the excitation power dependence of the anti-Stokes PL emission and compare it to the Stokes PL (Fig. S3). We record aS PL spectra beside NPoM cavities across a broad range of excitation powers P = 2 - 440 µW using a 785 nm CW excitation laser (Fig. S3a). The anti-Stokes PL shows a slight superlinear scaling with $P^{1.17\pm0.01}$, without signature of saturation. A similar scaling $P^{1.20\pm0.02}$ is observed for aS PL spectra enhanced by NPoM cavities (Fig. S3b). We exclude any damage by recording the spectrum at the lowest power before and after the power series. Figure S3c shows the normalized spectra recorded on one NPoM cavity. With increasing laser power, the peak intensity of the bright exciton increases with respect to the dark exciton (yellow arrow). This is also observed in the Stokes PL spectra recorded with λ_l = 633 nm. A possible reason is local heating by the excitation laser which increases the thermal population of the bright exciton.[1] The Stokes PL scales linearly $P^{1.00\pm0.02}$ beside NPoM cavities (Fig. S3d) and is slightly superlinear $P^{1.07\pm0.03}$ when enhanced by NPoM cavities (Fig. S3e).

Overall, the power dependence is close to linear, as previously observed for phonon-assisted anti-Stokes PL.[2] We therefore exclude two-photon excitation as a dominant excitation channel of anti-Stokes PL. The slightly superlinear scaling most likely originates from local heating by the excitation laser. The power dependence also indicates that photodoping and the formation of charged excitons is not the dominant mechanism here. In that case we would expect an increase of the low energy peak with respect to the bright exciton with increasing laser power, as previously observed by Wang et al.[3]

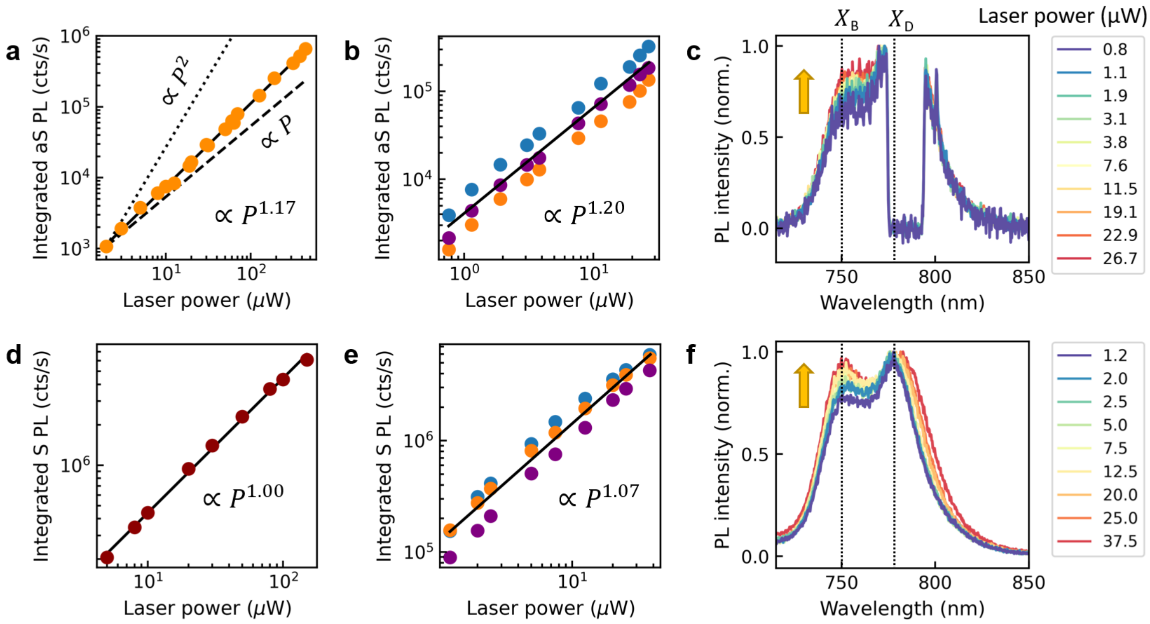

Figure S3: Excitation power dependence of anti-Stokes PL in WSe_2. (a) Anti-Stokes PL recorded beside NPoM cavities as function of CW excitation laser power. The PL intensity was integrated between 700-774 nm. Black solid line shows fit with exponent 1.17 ± 0.01. (b) Same as (a) but recorded on three NPoM cavities. Best fit with exponent 1.20 ± 0.02. (c) Normalized anti-Stokes PL spectra enhanced by NPoM cavity as function of laser power, see legend. Dashed lines show expected energies of bright X_B and dark X_D excitons. (a) – (c) measured with λ_l = 785 nm. (d) – (f) Excitation power dependence of Stokes PL recorded with λ_l = 633 nm. Fits are for exponent 1.00 ± 0.02 in (d) and 1.07 ± 0.03 in (e). The PL intensity was integrated between 700-820 nm. The peak ratio bright/dark increases with laser power, see arrows in (c) and (f).

Section S4: Estimate of local experimental enhancement factors by NPoM cavities

In our experiments we focus the excitation laser with a 100x, 0.9NA objective to a diffraction-limited laser spot with a diameter of (distance from centre where intensity drops to $1/e^2$):

$$d_l(\lambda_l) \approx 0.82\lambda_l/\mathrm{NA}. \tag{S1}$$

The laser spot is much larger than the diameter of the nanoparticle bottom facet where the enhancement occurs. From previous experiments we estimate the diameter of the nanoparticle bottom facet as $d_{\mathrm{cav}} \approx 0.4\, d_{\mathrm{NP}}$, where d_{NP} is the diameter of the nanoparticle.[4] Because of the size mismatch we also probe the PL emission from WSe_2 around the NPoM cavity. To estimate the enhancement at the nanoparticle bottom facet we calculate the local enhancement factor:

$$\mathrm{EF}_{\mathrm{loc}}(\lambda_l) \approx \mathrm{EF}_{\mathrm{exp}}(\lambda_l)\frac{d_l^2(\lambda_l)}{d_{\mathrm{cav}}^2}, \tag{S2}$$

where $\mathrm{EF}_{\mathrm{exp}}$ is the measured enhancement factor. For a nanoparticle diameter of 80 nm as used in our experiments we obtain $\mathrm{EF}_{\mathrm{loc}}(633\ \mathrm{nm}) \approx 300\,\mathrm{EF}_{\mathrm{exp}}(633\ \mathrm{nm})$ and $\mathrm{EF}_{\mathrm{loc}}(785\ \mathrm{nm}) \approx 500\,\mathrm{EF}_{\mathrm{exp}}(785\ \mathrm{nm})$.

Section S5: Estimate of PL efficiency and quantum yield

In the following, we estimate the PL efficiency in our experiments, which is the ratio of the number of PL photons collected by the objective lens to the number of photons illuminating the sample. Figure S4 compares an enhanced anti-Stokes PL spectrum (orange, from Fig. 1f) and reference spectrum (grey) with the backreflection of a 785 nm laser on a Au mirror (dark red). The laser intensity was attenuated by an OD6 notch filter and therefore is multiplied by 10^6. We use the laser backreflection to calibrate the detection efficiency of our setup by assuming that $\sim$100% of the 785 nm laser photons are reflected on the Au mirror and collected by the objective lens. By comparison of the peak areas of PL and laser, we estimate that $5 \cdot 10^{-4}$ % of the photons that illuminate the sample are upconverted with NPoM cavity, whereas $3 \cdot 10^{-6}$ % are upconverted without any NPoM cavity.

The quantum yield will be much higher, as not all incoming photons are coupled into the nanometer gap of the plasmonic cavity and absorbed by WSe_2, which is hard to quantify experimentally. We can however compare to an emitter with known quantum yield. The intensity of the enhanced upconverted PL ($\lambda_l = 785$ nm) is comparable to the intensity of Stokes PL ($\lambda_l = 633$ nm) of WSe_2 on glass (compare Fig. S4 orange and red, peak area ratio 0.95). The efficiency of the enhanced upconverted PL is therefore only approximately $\times 2$ smaller than that of the Stokes PL on glass (accounting for $\sim$50% undetected emission into the glass). From literature, the quantum yield of unprocessed exfoliated WSe_2 at room temperature is known to be on the order of 1 %.[5] This suggests a similar quantum yield of 0.5 % for the enhanced upconverted PL, but uncertainties arise from the unknown ratio of absorption/emission, which can be different because of the different excitation wavelengths (785 nm instead of 633 nm), and the plasmonic cavity. Based on FDTD simulations we expect that the plasmonic cavities mostly enhance the absorption channel, because of the activation of the dark exciton (see Table S1). We therefore estimate a quantum yield of the enhanced upconverted PL on the order of 0.1 %.

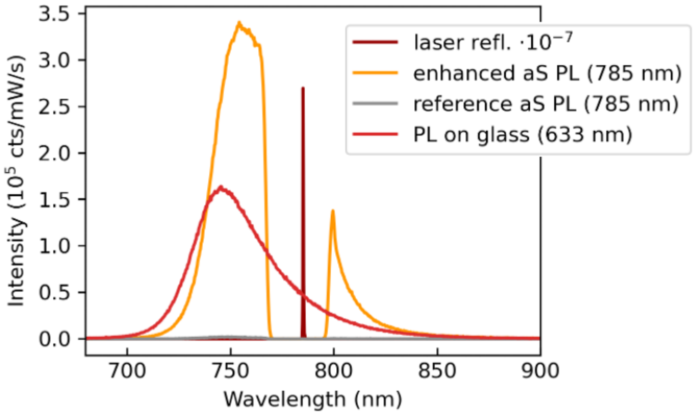

Figure S4: Estimate of PL efficiency and quantum yield. Comparison of anti-Stokes PL spectrum (orange) and reference spectrum (grey) from Fig. 1f to backreflection of 785 nm laser on Au mirror (dark red). Stokes PL spectrum for 633 nm laser excitation of WSe_2 on glass from Fig. S1 is shown in red.

Section S6: Statistics of dark exciton activation by plasmonic cavities and peak fits

To extract the relative strength of the dark and bright exciton PL emission, we fit the Stokes PL spectra recorded on all individual NPoM cavities. To find the lineshape of an individual exciton resonance at room temperature, we record the Stokes PL spectrum of $MoSe_2$ on a similar substrate without plasmonic cavities (Fig. S5a). The PL lineshape is best fit with a Voigt profile with equal weight of the Lorentzian and Gaussian contributions. We use the same lineshape to analyse the PL spectra of WSe_2. Figure S5b shows a PL spectrum of WSe_2 where the dark exciton is activated by an NPoM cavity. The spectrum is fit with two Voigt peak components of equal FWHM. The asymmetric PL lineshape of WSe_2 recorded beside the plasmonic cavities is also well explained by a fit with two Voigt peak components (Fig. S5c). For the fit, we fix the spectral separation of the two components to that in Fig. S5b (56 meV) and use the same FWHM for both components.

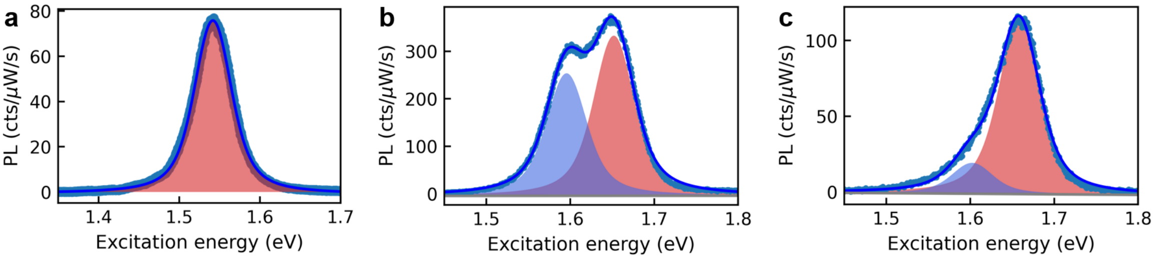

Figure S5: Peak fits of Stokes PL emission. (a) Fit of PL emission of $MoSe_2$ with Voigt lineshape. (b) Fit of PL emission of WSe_2 enhanced by NPoM with two Voigt peaks to fit dark (blue) and bright (red) excitons. (c) Same as (b) but for PL emission recorded beside NPoM.

We use similar peak fits to analyse all Stokes PL spectra from the statistical measurements on NPoM and NDoM cavities. Figure S6a shows the variation of bright and dark exciton wavelengths from the Stokes PL spectra enhanced by NPoM cavities, from which we obtain $\lambda_{\mathrm{B}} = 749 \pm 3\ \mathrm{nm}$, $\lambda_{\mathrm{D}} = 775 \pm 5\ \mathrm{nm}$ and an energy difference $\Delta E_{\mathrm{DB}} = 55 \pm 8\ \mathrm{meV}$.

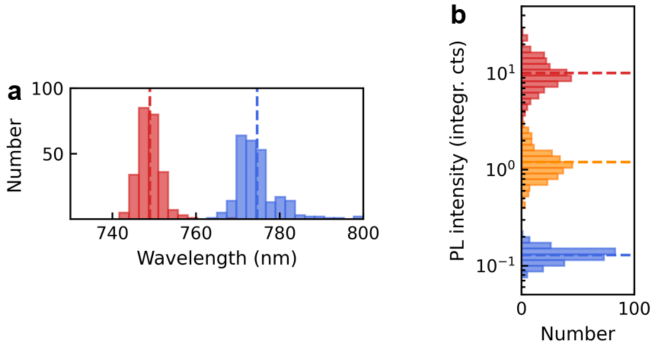

Figure S6: Statistics from fits of Stokes PL emission spectra. (a) Wavelengths of bright (red) and dark (blue) excitons from fit of 252 PL spectra enhanced by NPoM cavities. (b) PL intensity from 252 reference spectra recorded 2 µm beside each NPoM cavity. The histograms show the peak areas of the Stokes PL emission of the dark exciton (blue) and bright exciton (red) for excitation with $\lambda_l = 633$ nm, as well as the integrated PL cts of the anti-Stokes emission for excitation with $\lambda_l = 785$ nm (orange). Dashed lines show mean values.

To assess the spatial variation in PL intensity of WSe_2 on the Au substrate, we evaluate the integrated PL intensity from 252 reference spectra recorded 2 µm away from the NPoM cavities (Fig. S6b). The PL intensities of bright excitons (Stokes PL, red), anti-Stokes PL (orange) and dark excitons (Stokes PL, blue) each differ by one order of magnitude. The intensities of the bright exciton and anti-Stokes PL vary approximately by a factor of 2 and the dark exciton PL by 1.5 (FWHM of histograms in Fig. S6b). This explains part of the variations of the local enhancement factors shown in Fig. 5e.

Finally, we compare the dark exciton activation by NPoMs *vs* NDoMs (Fig. S7a). For NPoMs only 81 out of 252 cavities activate the dark exciton with $\mathrm{EF_D/EF_B} \geq 2$, while the activation is much more consistent for NDoMs where 154 out of 173 cavities activate the dark exciton (blue data points in Fig. S7a). As discussed in the main text, this also plays a key role for the enhancement of the anti-Stokes PL emission $\mathrm{EF_{aS}}$ (Fig. S7b). $\mathrm{EF_{aS}}$ is much more consistent for NDoMs (Fig. S7b, right) than for NPoMs (Fig. S7b, left). The enhancement factors of NDoMs are comparable to the enhancement of the subset of NPoMs with $\mathrm{EF_D/EF_B} \geq 2$ (compare blue data points in Fig. S7b).

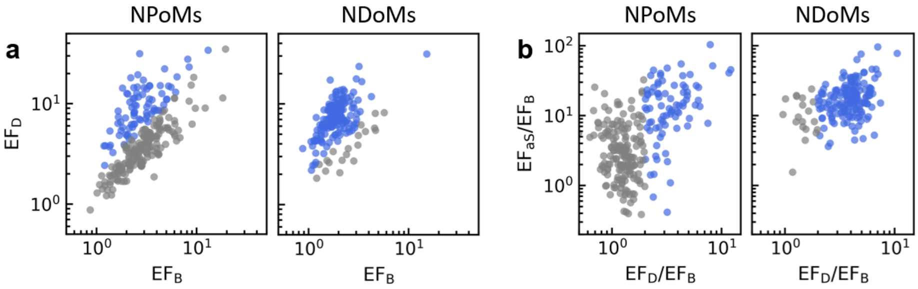

Figure S7: Statistics of dark exciton activation and anti-Stokes PL. (a) Enhancement of dark exciton $\mathrm{EF_D}$ vs enhancement of bright exciton $\mathrm{EF_B}$ in Stokes PL spectra recorded with $\lambda_l = 633$ nm for NPoMs (left) vs NDoMs (right). Blue data points are for cavities that activate the dark exciton $\mathrm{EF_D/EF_B} \geq 2$ and grey for $\mathrm{EF_D/EF_B} < 2$. (b) Relative strength of anti-Stokes PL enhancement $\mathrm{EF_{aS}}$ vs activation of dark exciton $\mathrm{EF_D}$ for NPoMs (left) vs NDoMs (right).

Section S7: FDTD simulation of PL enhancement

S7-1 Geometry of plasmonic NPoM cavities in FDTD – reproducing scattering spectra

We simulate the PL enhancement with the software package Lumerical FDTD Solutions from Ansys. We first identify a geometry of the NPoM cavities that is representative for our experiments (Fig. S8a). We use a spherical nanoparticle with $d_{\mathrm{NP}} = 80$ nm diameter, which is the mean diameter of the nanoparticles employed. The sphere is truncated at the bottom to obtain a spherical bottom facet with $0.4d_{\mathrm{NP}}$ diameter, which was identified as a representative geometry in previous simulations and experiments.[4] For the gap material we assume that the WSe_2 is encapsulated between two layers of molecules, i.e. citrate ligand molecules at the nanoparticle interface and polymer transfer residues between the Au substrate and WSe_2. These two layers are modelled with a constant refractive index of $n = 1.5$. For WSe_2 we use a film with thickness of 0.65 nm with the complex in-plane refractive index measured by Gu et al. for a monolayer,[6] and a constant and real out-of-plane refractive index of 2.72 as calculated by Laturia et al. from first principles.[7] In the simulation of emission enhancement we use a constant and real in-plane refractive index of 4.3 to avoid point-dipole emission into a lossy medium. We use a mesh override region with 0.4 nm in all directions within the NPoM gap, and 1 nm across the nanoparticle.

We simulate the scattering spectra by illuminating the NPoM cavity with a total-field scattered-field (TFSF) plane-wave source either from the side or top (Fig. S8b, c). The scattered light is detected with a power monitor around the TFSF source. The experimentally measured dark field spectra are best reproduced when assuming a thickness of 0.75 nm for the molecular layers around WSe_2 (compare Fig. S8b blue with Fig. 1d red). The spectrum for side illumination is most representative for the experiments as the experimental dark-field illumination is at 64–75° incidence angle.[4]

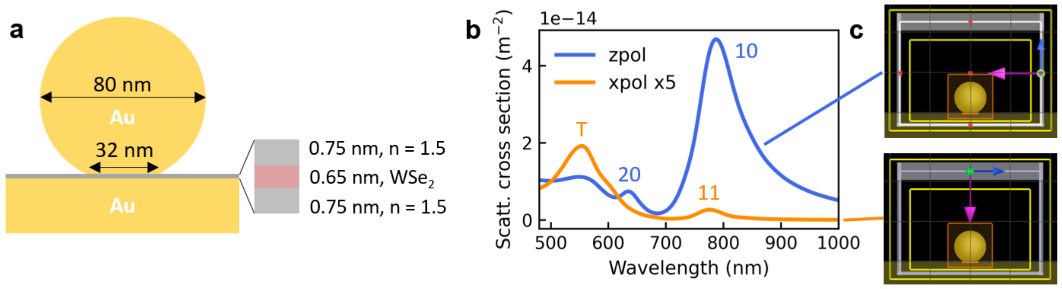

Figure S8: NPoM geometry for FDTD simulations by reproducing experimental scattering spectra. (a) Geometry of NPoM cavity used in FDTD simulations. (b) Simulated scattering cross section by excitation from the side (blue) or top (orange) with a plane-wave source. Plasmonic modes are labelled as in Ref. 4. (c) Simulation geometries for side- and top-illumination. Gray box is TFSF source with pink arrow showing propagation direction and blue arrow polarization of E field. Yellow boxes are power monitors to detect the absorbed and scattered power. Orange boxes show mesh-override regions.

S7-2 Photoluminescence enhancement of NPoMs

The photoluminescence enhancement has contributions from an excitation enhancement $\mathrm{EF}_{exc}(\lambda_l)$ and an emission enhancement $\mathrm{EF}_{em}(\lambda_{em})$,

$$\mathrm{EF}_{\mathrm{PL}}(\lambda_l, \lambda_{em}) = \mathrm{EF}_{exc}(\lambda_l)\mathrm{EF}_{em}(\lambda_{em}). \tag{S3}$$

We calculate the excitation enhancement from the local electric fields at the WSe_2 layer in the plasmonic cavity

$$\mathrm{EF}_{exc}(\lambda_l) = \frac{\iint_{xy} dxdy\, |E(x,y,\lambda_l)|^2}{\iint_{xy} dxdy\, |E_0(x,y,\lambda_l)|^2}, \tag{S4}$$

where E is the electric field amplitude in the presence of the plasmonic nanostructure and E_0 the amplitude without the plasmonic nanostructure. The integration is carried out over the size of the laser excitation spot. We implement this in Lumerical FDTD by using a Gaussian beam source that approximates the full vectorial fields of a focussed beam with a numerical aperture of 0.9 (Fig. S9a). The enhanced electric field $E(x,y,\lambda_l)$ is detected with an E field monitor in the middle of the TMD layer and E_0 is recorded with the same simulation layout but without the nanoparticle. Figure S8b shows the average field enhancement in a circular area with diameter 50 nm below the nanoparticle bottom facet. The enhancement is largest EF_{exc}(785 nm) = $1{\cdot}10^3$ at the excitation wavelength for anti-Stokes PL and smaller EF_{exc}(633 nm) = $8{\cdot}10^1$ at the excitation wavelength for Stokes PL. The field enhancement is strongly localized to the nanoparticle bottom facet (Fig. S9c, top). Fringing of the field lines near the edges of the nanoparticle bottom facet leads to in-plane field enhancement (Fig. S9c, bottom and Fig. 1b). The spatial field profiles in the NPoM gap have a node at the center, which is expected for excitation of the 11 and 21 plasmonic modes, which couple to far field radiation with in-plane polarization (Fig. 1b, and S9b, insets).[4] To estimate the enhancement measured in our experiments, we average the local enhancement over the size d_l of the laser spot, assuming that the field enhancement is 1 outside the 50 nm circular area shown Fig. S8b. We obtain EF_{exc}(633 nm) = 2.8 for Stokes PL and EF_{exc}(785 nm) = 19 for anti-Stokes PL.

The emission enhancement is given by

$$\mathrm{EF}_{em}(\lambda_{em}) = \frac{\iint_{x,y} dxdy\, P(x,y,\lambda_{em})}{\iint_{x,y} dxdy\, P_0(x,y,\lambda_{em})}, \tag{S5}$$

where $P(x,y,\lambda_{em})$ is the emitted power of a dipole in the NPoM gap, and P_0 the emitted power without the NPoM. We simulate this by placing an array of point dipoles in the NPoM gap and record the radiated power with a monitor above the substrate (Fig. S9d). The lateral size of the monitor is chosen to collect light with emission angles up to 64°, as for the 0.9 NA objective used in the experiments. We use a circular dipole array with 50 nm diameter and 2 nm lattice constant to sample the enhancement in the NPoM gap. To avoid destructive interference, we position the dipoles only in one half of the NPoM gap. This is justified as the exciton Bohr radius r_{ex} < 2 nm is much smaller than the lateral extension of the NPoM gap d_{cav} = 32 nm preventing coherent emission beyond several nm distance. The emission enhancement for out-of-plane dipole orientation is much larger than for in-plane orientation (Fig. S9e, compare blue and orange). This is because the electric fields in the NPoM gap are primarily polarized out-of-plane. We obtain an emission enhancement EF_{em}(775 nm) = $5{\cdot}10^3$ for the dark exciton with out-of-plane transition dipole and EF_{em}(750 nm) = $2{\cdot}10^1$ for the bright exciton with in-plane transition dipole. When averaging over the size of the laser spot the enhancement measured in experiments is EF_{em}(775 nm) = 19 and EF_{em}(750 nm) = 1.08.

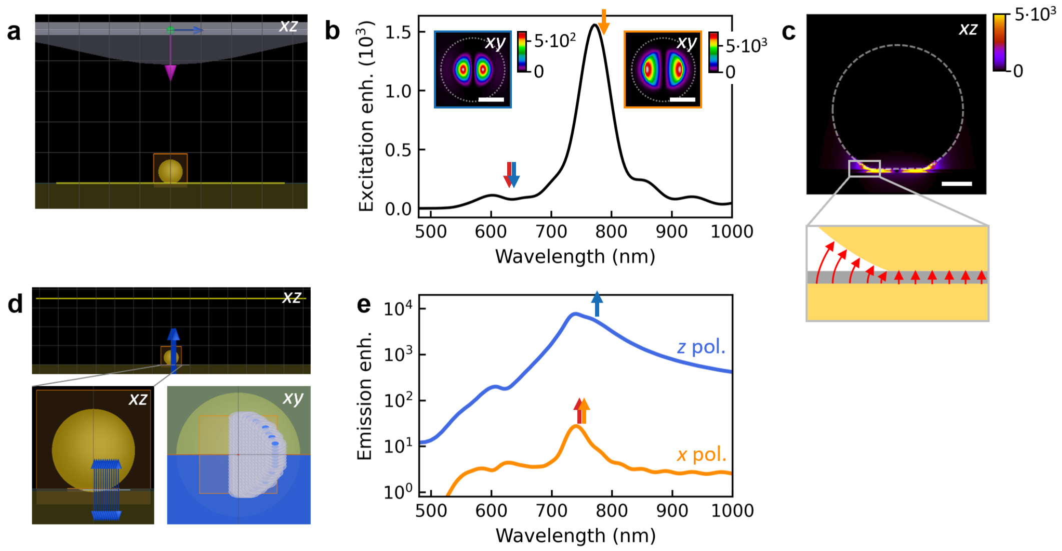

Figure S9: FDTD simulation of excitation and emission enhancement by NPoM cavity. (a) Layout for simulation of excitation enhancement with Gaussian beam source (top) and electric field monitor (bottom, yellow). (b) Average electric field intensity enhancement within circular area with 50 nm diameter (dotted lines in insets) below nanoparticle bottom facet. Insets show the field intensity enhancement at $\lambda_l = 633$ nm, left, and $\lambda_l =$ 785 nm, right. Arrows show excitation wavelengths for Stokes PL (blue, red) and anti-Stokes PL (orange). (c - top) E field intensity enhancement for cross section through nanoparticle. Dashed line shows outline of nanoparticle. (c - bottom) Sketch of electric field direction at edge of bottom facet. (d) Layout for simulation of emission enhancement. A dipole array with diameter of 50 nm (blue arrows) is placed below nanoparticle bottom facet and the emitted power is detected with a monitor (top, yellow). Blue area shows symmetry plane used in simulation. (e) Emission enhancement for dipole orientation along *z* (blue) and *x* (orange). Arrows show the emission wavelengths of the dark (blue) and bright (orange) excitons. Scale bars are 20 nm.

The overall enhancement factors $\mathrm{EF_{PL}}(\lambda_l, \lambda_{em})$ are summarized in Table S1. The simulated enhancement of the bright exciton Stokes and anti-Stokes PL agree well with the measured average enhancement factors. The enhancement of the dark exciton, on the other hand, is overestimated, which could be caused by a less ideal collection efficiency for high-angle emission in the experiments than in the simulations, and by an overestimation of the dark exciton intensity in the reference spectra. If we assume that anti-Stokes PL instead occurs through the excitation of the bright exciton with in-plane transition dipole we obtain an enhancement $\mathrm{EF_{PL,aS}}$ = 1.6, much smaller than in the experiments. This highlights the role of the dark exciton as an excitation channel.

	**Stokes, bright**	**Stokes, dark**	**Anti-Stokes, bright**
$\mathbf{EF_{exc}}$	2.8 ($8\cdot10^1$)	2.8 ($8\cdot10^1$)	19 ($1\cdot10^3$)
$\mathbf{EF_{em}}$	1.08 ($2\cdot10^1$)	18 ($5\cdot10^3$)	1.08 ($2\cdot10^1$)
$\mathbf{EF_{PL}}$	**3.0** ($2\cdot10^3$)	**50** ($4\cdot10^5$)	**21** ($2\cdot10^4$)
$\mathbf{EF_{exp}}$	**3**	**7**	**20**

Table S1: PL enhancement of NPoM cavity from FDTD. $\mathrm{EF_{exc}}$ excitation enhancement, $\mathrm{EF_{em}}$ emission enhancement, and $\mathrm{EF_{PL}}$ overall PL enhancement of bright exciton (red) and dark exciton (blue) in Stokes PL, and bright exciton in anti-Stokes PL (orange). Values are average enhancement over size of diffraction-limited illumination spot, while values in brackets are average enhancement over area with 50 nm diameter at the nanoparticle bottom facet. Last row states measured average enhancement factors $\mathrm{EF_{exp}}$.

While Au is the most stable plasmonic material, our approach also carries over to other lower-cost plasmonic materials, such as Ag and Cu. Figure S10 shows the simulated excitation enhancement, as calculated in Fig. S9b, when replacing Au with Ag or Cu. Ag provides even larger excitation enhancement, while the enhancement by Cu is slightly lower. The plasmonic resonances for Ag and Cu NPoMs are blue shifted with respect to Au. Slightly larger nanoparticle sizes would be therefore required for a resonant enhancement of the WSe_2 excitons.

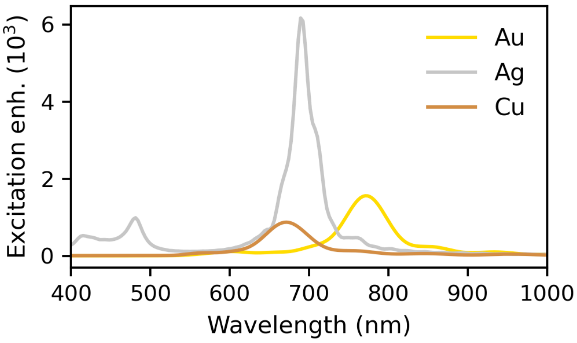

Figure S10: FDTD simulations of the excitation enhancement by NPoM cavities consisting of Au, Ag, and Cu, showing average field intensity enhancement below the bottom facet of the nanoparticle, in a circular area with diameter of 50 nm (see Fig. S9b). All other parameters are the same as in Fig. S9.

S7-3 Photoluminescence enhancement of NDoMs

Using a similar approach as for NPoMs, we simulate the PL enhancement by nanodecahedra-on-mirror (NDoM) cavities. We reconstruct the geometry of NDoMs with 70 nm edge length in Lumerical FDTD (Fig. S11a). The scattering spectra show two dominant resonances between 700 and 800 nm, as observed experimentally (compare Fig. S11b with Fig. 4c). The excitation enhancement increases with wavelength and peaks close to λ_l = 1000 nm at a plasmonic mode that is similar to the 11 mode of the NPoM, which couples best to a Gaussian excitation spot (Fig. S11c). At λ_l = 633 nm and 785 nm the electric field enhancement is located close to the edges of the nanoparticle bottom facet (Fig. S11c, insets). For the emission enhancement, we run two simulations with dipole arrays on the left half and the right half of the bottom facet, and calculate their average. The emission enhancement for in-plane dipoles has a resonance at 760 nm, close to the emission wavelength of the bright exciton (Fig. S11d). Similar to NPoMs the emission enhancement is more than one order of magnitude larger for dipoles with out-of-plane orientation which leads to a larger enhancement of the dark exciton.

The simulated enhancement factors for the NDoM cavity are reported in Table S2. While there is good agreement with experiment for the Stokes PL enhancement of the bright and dark exciton, the average enhancement of the anti-Stokes PL is underestimated by the simulations. A possible reason is destructive interference in the simulations of the emission enhancement. The plasmonic near-field of the NDoM cavities flips its direction at more places than for NPoM cavities (compare insets of Fig. S11c with S9b), which leads to cancellations within the area of the dipole arrays in our simulations. Furthermore, our simulations do not account for the spatial overlap of the excitation and emission enhancement (see Eqs. S3-S5) which is better for NDoMs where both in-plane and out-of-plane field enhancement are located at the edges of the bottom facets, while for NPoMs the out-of-plane field enhancement occurs close to the center of the bottom facet. Accounting for these effects requires a theory that accounts for exciton delocalization and diffusion in the definition of the enhancement factors, which is beyond the scope of this work.

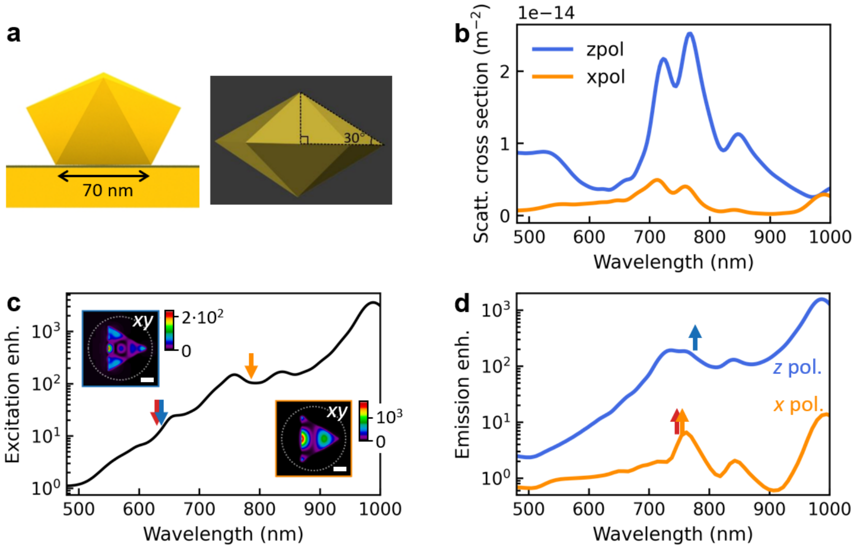

Figure S11: FDTD simulation of PL enhancement by NDoM cavities. (a) Geometry of an NDoM cavity as used in the simulations. (b) Simulated scattering cross section for excitation from the side (blue) and top (orange) as in Fig. S8b, c. (c) Average electric field intensity enhancement within circular area with 100 nm diameter (dotted lines in insets) below nanoparticle bottom facet. Insets show the field intensity enhancement at $\lambda_l = 633$ nm, left, and $\lambda_l = 785$ nm, right. Arrows show excitation wavelengths for Stokes PL (blue, red) and anti-Stokes PL (orange). Scale bars are 20 nm. (d) Emission enhancement for dipole orientation along *z* (blue) and *x* (orange). Arrows show the emission wavelengths of the dark (blue) and bright (orange) excitons.

	**Stokes, bright**	**Stokes, dark**	**Anti-Stokes, bright**
$\mathbf{EF_{exc}}$	1.9 (1·10^1)	1.9 (1·10^1)	5 (1·10^2)
$\mathbf{EF_{em}}$	1.1 (5·10^0)	3.0 (2·10^2)	1.1 (5·10^0)
$\mathbf{EF_{PL}}$	**2.1** (5·10^1)	**5.7** (2·10^3)	**5.5** (5·10^2)
$\mathbf{EF_{exp}}$	**2**	**7.5**	**44**

Table S2: PL enhancement of NDoM cavity from FDTD. $\mathrm{EF_{exc}}$ excitation enhancement, $\mathrm{EF_{em}}$ emission enhancement, and $\mathrm{EF_{PL}}$ overall PL enhancement of bright exciton (red) and dark exciton (blue) in Stokes PL, and bright exciton in anti-Stokes PL (orange). Values are average enhancement over size of diffraction-limited illumination spot, while values in brackets are average enhancement over area with 100 nm diameter at the nanoparticle bottom facet. Last row states measured average enhancement factors $\mathrm{EF_{exp}}$.

Section S8: Electrochemical gating of PL emission

We observe a modulation of the PL emission intensity through electrochemical gating, both for $\lambda_l =$ 785 nm (Fig. S12) and $\lambda_l =$ 633 nm (Fig. S13). Fig. S12a shows a cyclic voltammogram with a scan rate of 20 mV/s, which is representative of the entire sample, of which only some areas are covered with WSe_2. No significant faradaic electrochemical currents were detected during the cyclic voltammetry measurements other than weak capacitive charging of the thin liquid layer. The PL intensity recorded with $\lambda_l = 785\ \mathrm{nm}$ is significantly modulated during the potential cycling (Fig. S12b). The spectra in Fig. 5b are calculated from this scan using the spectra between 0 s and 32 s. Figure S12c,d show the intensity modulation of the anti-Stokes and Stokes PL during chronoamperometric switching of the potential between +0.2 V and -0.4 V (Fig. S12e). The intensities recorded beside the NDoM cavity are shown in grey. The enhancement of the PL is not as large as outside the electrochemical cell, as it is harder to focus high-angle light into the cell, which is needed for efficient excitation of the dark exciton. The modulation of the enhanced PL is almost as large as in the much weaker spectra recorded beside the cavity (grey), which shows that ions can penetrate into the NDoM gap. A slight drop in PL intensity during the modulation occurs due to sample drift out of the laser focus.

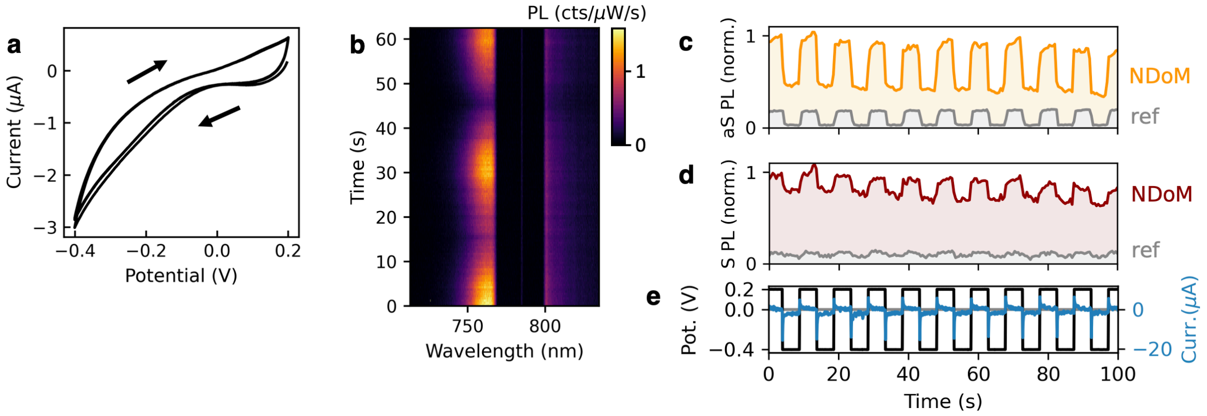

Figure S12: Electrochemical gating of PL emission with $\lambda_l = 785\ \mathrm{nm}$. (a) Cyclic voltammetry (CV) curve with three cycles, sweeping potential between 0.2 V and -0.4 V with a scan rate of 20 mV/s. (b) PL intensity during the second and third cycle in (a), enhanced by NDoM cavity. (c)-(e) Modulation of PL intensity by step-wise switching potential between 0.2 V and -0.4V. (c) Integrated intensity on anti-Stokes side enhanced by NDoM cavity (orange) and beside cavity (grey). (d) Integrated intensity on Stokes side enhanced by NDoM cavity (red) and beside cavity (grey). (e) Current (blue) and voltage (black) during switching. Data in (c)-(e) were subsequently recorded.

Figure S13 shows the electrochemical modulation of PL emission excited with $\lambda_l = 633\ \mathrm{nm}$. The PL emission enhanced by an NDoM cavity is strongly modulated (Fig. S13a) during step-wise chronoamperometric switching of the potential between +0.2 V and -0.4 V. Average spectra for the two potentials are shown in Fig. S13b, together with fits to two Voigt peaks thus giving the bright and dark exciton intensities (Fig. S13c) and wavelengths (Fig. S13d). The intensity modulation of the bright exciton is larger than that of the dark exciton. The data in Fig. 13c are used to calculate the dark/bright ratio in Fig. 5d. The dark and bright exciton emission wavelengths are both modulated by $\Delta\lambda_{PL} = 2$ nm with a red shift at negative potentials.

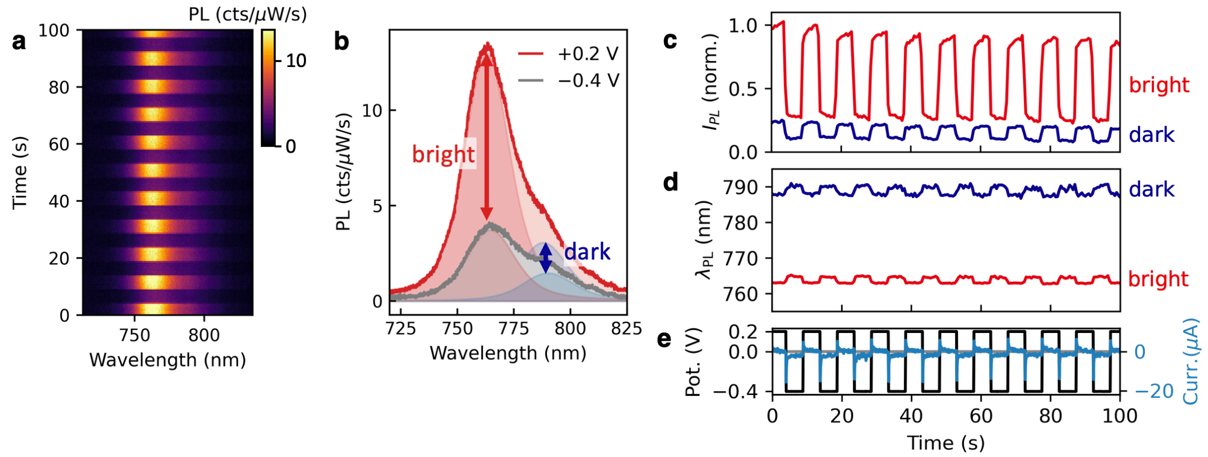

Figure S13: Electrochemical gating of PL emission with $\lambda_l = 633$ nm. (a) Modulation of Stokes PL emission enhanced by NDoM cavity while step-wise switching potential between +0.2 V (bright) and -0.4 V (dim), see (e) for time-dependent voltage. (b) Average PL spectra for the two potentials with fit of bright and dark exciton, see red and blue components. (c) Modulation of bright and dark exciton intensity and (d) emission wavelengths, while (e) step-wise switching potential (black) and el. current (blue).

1. Zhang X-X, You Y, Zhao SYF, Heinz TF. Experimental Evidence for Dark Excitons in Monolayer WSe_2. *Phys Rev Lett* **115**, 257403 (2015).
2. Jones AM*, et al.* Excitonic luminescence upconversion in a two-dimensional semiconductor. *Nat Phys* **12**, 323-327 (2016).
3. Wang Z*, et al.* Nanocavity-induced trion emission from atomically thin WSe2. *Sci Rep* **12**, 15861 (2022).
4. Elliott E*, et al.* Fingerprinting the Hidden Facets of Plasmonic Nanocavities. *ACS Photonics* **9**, 2643-2651 (2022).
5. Kim H*, et al.* Synthetic WSe2 monolayers with high photoluminescence quantum yield. *Sci Adv* **5**, eaau4728 (2019).
6. Gu H*, et al.* Layer-dependent dielectric and optical properties of centimeter-scale 2D WSe2: evolution from a single layer to few layers. *Nanoscale* **11**, 22762-22771 (2019).
7. Laturia A, Van de Put ML, Vandenberghe WG. Dielectric properties of hexagonal boron nitride and transition metal dichalcogenides: from monolayer to bulk. *npj 2D Mater Appl* **2**, 6 (2018).
